# Machine Learning Techniques for Blind Beam Alignment in mmWave Massive MIMO

**DOI:** 10.3390/e26080626

**Published:** 2024-07-25

**Authors:** Aymen Ktari, Hadi Ghauch, Ghaya Rekaya-Ben Othman

**Affiliations:** Télécom Paris, 91120 Paris, France; hadi.ghauch@telecom-paris.fr (H.G.); ghaya.rekaya@telecom-paris.fr (G.R.-B.O.)

**Keywords:** mmWave MIMO, massive antennas, ML-based Beam Alignment, blind BA, Matrix Factorization, Multi-Layer Perceptron, non-linear regression

## Abstract

This paper proposes methods for Machine Learning (ML)-based Beam Alignment (BA), using low-complexity ML models, and achieves a small pilot overhead. We assume a single-user massive mmWave MIMO, Uplink, using a fully analog architecture. Assuming large-dimension codebooks of possible beam patterns at UE and BS, this data-driven and model-based approach aims to partially and blindly sound a small subset of beams from these codebooks. The proposed BA is blind (no CSI), based on Received Signal Energies (RSEs), and circumvents the need for exhaustively sounding all possible beams. A sub-sampled subset of beams is then used to train several ML models such as low-rank Matrix Factorization (MF), non-negative MF (NMF), and shallow Multi-Layer Perceptron (MLP). We provide an extensive mathematical description of these models and the algorithms for each of them. Our extensive numerical results show that, by sounding only 10% of the beams from the UE and BS codebooks, the proposed ML tools are able to accurately predict the non-sounded beams through multiple transmitted power regimes. This observation holds as the codebook sizes at UE and BS vary from 128×128 to 1024×1024.

## 1. Introduction

Driven by the explosive growth trend of large-scale connectivity and higher data rate systems, wireless data traffic is expected to exponentially increase, growing to 5 zettabytes per month and reaching a 100 Gps data rate by 2030 [1] Thus, the latency in the 6th Generation is predicted to reach 0.1 ms, representing 10% of 5G latency, in order to support new emerging technical needs, including holographic images, Internet of Things applications, and autonomous driving.

Beam Alignment is frequently defined in the literature as beam sounding, i.e., beam training. It illustrates a fundamental problem in millimeter-wave Multiple Input, Multiple Output systems, defined as the exchange of information between the user equipment UE and the base station BS in order to accurately select the optimal beam-steering direction. The process of aligning the beams is related to several technical problems, such as beam forming, beam sweeping, beam tracking, and beam selection. The whole framework that unites these operations between UE and BS is often denoted as the Beam Management. To fulfill the BA task, beam patterns stored in large codebooks are used at both UE and BS. In fact, pencil beams with directional gain are increasingly being used in several applications in order to alleviate the severe path-loss attenuation and increase capacity and data throughput. On the other hand, massive MIMO systems provide large gain in spectral and energy efficiencies compared with conventional MIMO systems. Using mmWave technology, these systems mainly offer a better communication quality by increasing the system bandwidth and reducing the effects of noise and interference. Due to the diversification of future 5G and towards 6G applications and intelligent systems, scientists predict the continuous generation of massive datasets for deep processing through large bandwidths, which introduces mmWave bands as the golden spectrum band candidates. However, the limitations of mmWave communication physical properties of the channel are crucial: scattering, attenuation, low coherence time related to the Doppler effect, penetration loss, environmental constraints, and complex channel modeling in realistic urban scenarios. The major problem we aim to encounter in this paper is the inevitable high signaling/training *overhead*. For this reason, the main trade-off is to browse the most accurate and the least complex ML algorithm that optimizes finding the optimal beam pair based on sounded instantaneous Received Signal Energies and using the minimum (possible) amount of training samples.

**Contributions:** In this current work, we propose ML-based BA methods, for a single user massive mmWave MIMO, Uplink, with a wide-band channel. We assume a single radio frequency chain with large codebooks of possible analog beams at BS (also known as BS codebook) and UE (also known as UE codebook). We define a beam pair as one beam from the BS and UE codebook. By approximating the SNR with the Receive Signal Energy (RSE), we bypass the need for CSI, i.e., a blind approach. We sub-sample large codebooks into smaller sub-sampled BS and UE codebooks, and sound the beam pairs from the sub-sampled codebooks to generate the training set—a novelty of the approach. Using the RSE of the sounded beam pairs (sub-sampled codebooks), we propose to train the following ML methods to predict the RSE of the beam pairs that were not sounded: Matrix Factorization (MF), non-negative Matrix Factorization (NMF), and feed-forward (shallow) Multi-Layer Perceptron (MLP).

- We formulate the MF and NMF problems. We propose to use Block Coordinate Descent (BCD) and Block Gradient Descent (BGD) methods to solve each problem. We derive in depth all the update equations for these methods. We show that the BCD method converges to a stationary point from both MF and NMF problems. Our extensive numerical results show that, sub-sampling 10% of the BS/UE codebooks, the remaining RSE values can be predicted extremely well (with a training/test error $\approx 10^{-6}$) for every antenna configuration.
- We develop at length the equations of a general MLP model, the resulting loss function, and the corresponding optimization problem. In addition, we derive the equations of back-propagation for the MLP in question. Using extensive numerical results, we observe that sounding 10% of original codebooks is sufficient to predict the RSE of the beam pairs that were not sounded, with negligible training/test error.
- We numerically compare the training/test losses of all the proposed models for a varying cardinality of codebooks and transmit powers. These results suggest that the BCD method for MF/NMF outperforms the MLP in terms of training and test error. Meanwhile, BCD for MF/NMF has a large computational complexity and the MLP exhibits medium complexity.
- Interestingly, by sounding 10% of the BS/UE codebooks, the proposed ML models can predict the unknown RSE (beam pairs not sounded) with a negligible test error. Thus, the proposed methods achieve a 90% reduction in pilot signaling overhead, compared with the SotA benchmark, without any noticeable loss in performance.

**Notations:** Matrices and vectors are respectively written in boldface upper-case and lower-case letters. We use $Tr\left[A\right],A^{T},A^{-1},A^{H}{,|A|,||A||}_{F}$ for the trace, transpose, inverse, conjugate transpose, determinant, and Frobenius norm of a matrix A and the n×n identity matrix. ${\left[A\right]}_{i,j}$ is used to denote the (i, j)th entry of a matrix A. We denote the Hadamard product by ∘, while ${\left[a\right]}_{+}:=\max\left(a,0\right)$ illustrates a Euclidean projection of a on $R_{+}^{D}$ and is applied element by element on a. We denote |x| the absolute value of *x* and ${\left[x\right]}_{t}$ as the entry *t* of a vector x.

**Methods/Experiment:** The proposed approach is data driven and model based. The dataset is generated following the Saleh Valenzuela wide-band mmWave system model. It is based on Received Signal Energies for each and every beam pair in the massive MIMO Uplink setup stored in separate .csv files. The model-based solution to the empirical risk minimization includes deriving a closed-form solution to the formulated non-convex optimization problem, stating the theoretical guarantees of convergence and empirically illustrating the success of the proposed partial and blind Beam Alignment procedure using different algorithms. All simulations are executed on Infres GPU servers and the Comelec laboratory PC at Télécom Paris, having the following characteristics: Intel(R) Core(TM) i5-8365U CPU @ 1.60 GHz, 16 Go (RAM), x64 processor, and 64-bit operating system under the license of Windows 10 Enterprise LTSC 2018, version 1809. The manufacturer is Dell and is located in Paris, France. All python packages used in this work (numpy, scipy, keras, pytorch, matplolib..) are related to python 3.9 release. In fact, the experimental protocol is based on offline grid-search cross-validation, which requires GPU processing for the selection of optimal hyperparameters and online training/prediction for Matrix Factorization, non-negative Matrix Factorization, and Multi-Layer Perceptron. The comparison is conducted following a Quality of Service-based approach, simulating a variety of MIMO configurations and architectural setups, investigating the impact of varying the Received Signal Energy regime and empirically stating intersections and differences in the impact of the transmit power on model behaviors, loss values, optimal signaling overhead ratio, and optimal hyperparameters.

- Problem Statement: The main challenge addressed in this study is the high signaling overhead in Beam Alignment for mmWave MIMO systems, which hampers the efficient selection of optimal beam-steering directions.
- Research Questions and Hypotheses: This study investigates whether machine learning methods can effectively reduce the signaling overhead required for accurate beam-pair prediction in mmWave MIMO systems.
- Objectives and Aims: The primary objective is to develop and evaluate ML-based BA methods that minimize the training overhead while maintaining high accuracy in predicting the RSE for unsounded beam pairs.
- Significance and Rationale: The study proposes a novel approach to BA using ML techniques, which can lead to a substantial reduction in pilot signaling overhead and enhance the efficiency of future wireless communication systems.

## 2. Literature Survey

In conventional standards, *Exhaustive* BA, also called Brute Force BA, is the de facto approach for the alignment process. It is based on sounding all available beams at both UE and BS codebooks in order to exhaustively select the optimal beam pair. One obvious drawback is the fact that the resulting signaling overhead scales as the product of the UE and BS codebook sizes. At 60 GHz, the Exhaustive BA has been adopted in several mmWave WLAN or WPAN communication technologies, e.g., IEEE 802.15.3c [2] and IEEE 802.11ad [3]. It is conventionally applied in small MIMO configurations using small codebook sizes (e.g., codebooks of size 8×8 for LTE) and guarantees optimal performance. For cellular networks [4], V2X communications, Unmanned Aerial Vehicles, or High-Speed Train applications, the infeasibility of brute-force-based BA pushes scientists to reduce the large signaling overhead from using massive antennas systems. State-of-the-art methods can be divided into two categories: classic BA and learning-based BA. Traditional techniques tend to use a more and more structured Beam Alignment design such as hierarchical multi-level codebooks [5] (training beamforming vectors are constructed with different beam widths at different levels) and an overlapped beam pattern [6], where the main idea is to augment the amount of information carried by each channel measurement, reducing the required channel estimation time and beam coding [7], where we assign a unique code signature to each beam angle in addition to subspace estimation/decomposition-based BA [8]. Compressed sensing-based algorithms [9] are also used in this context, taking advantage of channel sparsity. Therefore, we state two intersections in classic methods: they generally rely on CSI exchange and Exhaustive BA. In contrast, lately, Machine Learning (ML)-based BA has emerged and is continuously leading to some promising results. For instance, statistical models such as Kolmogorov model-based BA in [10] with sub-sampled codebooks reduce the signaling overhead: 15% of Exhaustive BA provides accurate predictions for optimal beams at UE and BS in a partial BA procedure, similar to our approach. Deep learning through shallow neural networks is increasingly used by Wireless Communication scientists, where we distinguish two major paradigms: first, the ML methods related to Supervised Learning (SL) via a Support Vector Machine and Multi-Layer Perceptrons for joint analog beam selection in [11], convolutional neural networks for beam management in sub-6 GHz in [12] and for calibrated beam training in [13], recurrent neural networks such as Long Short-Term Memory network for beam tracking in [14,15,16], auto-encoders for beam management in [17], and several other neural architectures, and second, Reinforcement Learning (RL) in [18,19,20], generally used to resolve the problems of Multi-Armed Bandit and Markov decision process. In addition, neural architectures have the ability to extract features from the hidden interactions between BS and UE, providing fast and accurate estimations through different MIMO setups and channel realizations, especially when applied to massive datasets where more and more data/train samples are embedded. This work is an extension of [21]. In this paper, we extend the channel model to wide-band and we add multiple RF-chains at BS in a fully analog low-complexity architecture, where we investigate more ML tools for partial and blind BA. This paper is one of the first attempts to apply MF/NMF models and shallow Multi-Layer Perceptrons to a blind and partial Beam Alignment for massive mmWave SU-MIMO. Our work in [22] is related to the same approach and objectives, where we quantize the output of each RF-chain.

## 3. System Model

**Figure 1:**
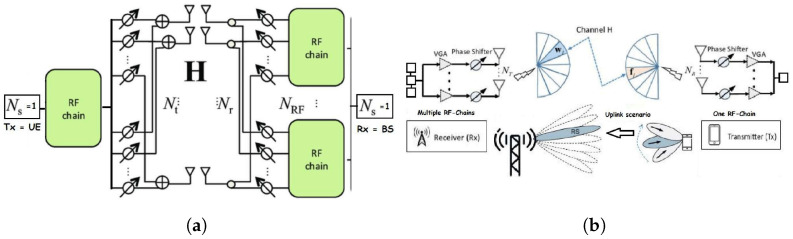
Proposed BA diagram representation: (**a**) fully analog MIMO architecture using a single RF chain at UE and multiple RF chains at BS; (**b**) simplified illustration of Beam Alignment problem.

In this section, we illustrate the mmWave MIMO point-to-point system model. We consider an Uplink transmission from multiple-antenna user equipment UE using a single radio frequency chain and a multiple-antenna base station BS using multiple radio frequency chains. The proposed ML methods are performed at the BS, which has higher computational resources than UE. Figure 1a,b provide a diagram representation of the proposed architecture. UE and BS are respectively equipped with Uniform Linear Arrays of $N_{T}$ and $N_{R}$ antenna. We propose a low-cost/complexity fully analog architecture where UE has one radio frequency chain and BS has $N_{rf}$ radio frequency chains. UE selects its analog beamformer $f_{u}\in C^{N_{T}}$ from a codebook of feasible beam choices, u∈T, where T is the corresponding index set. Moreover, BS selects its analog combiner $W_{i}\in C^{N_{R}\times N_{rf}}$ from a codebook i∈R with R as the index set of the codebook. We denote with $C_{T}$ the number of possible beamforming vectors at UE, i.e., the size/cardinality of the UE codebook, $\left|T\right|=C_{T}$ and $C_{R}$, and the size/cardinality of the BS codebook, $\left|R\right|=C_{R}$. Both beamforming and combining are fully performed in the analog domain using phase shifters at UE and BS; thus, they satisfy the following constant modulus constraints, $\forall \;r\in \left\{1,\ldots ,N_{R}\right\},\;\forall \;t\in \left\{1,\ldots ,N_{rf}\right\}$:

$$W_{i}\in C^{N_{R}\times N_{rf}}\;,\;\left|\;{\left[W_{i}\right]}_{r,t}\;\right|=\frac{1}{N_{rf}N_{R}}\;$$

$$f_{u}\in C^{N_{T}}\;,\;\left|\;{\left[f_{u}\right]}_{t}\;\right|=\frac{1}{N_{T}}\;,\;\forall \;t\in \left\{1,\ldots ,N_{T}\right\}$$

For our proposed approach, BS is responsible for receiving signal energies, denoted as RSE, in order to learn their patterns and features for the purpose of accurately predicting the optimal beam indexes from their corresponding codebooks and send them to UE. We adopt the wide-band channel model $G\in C^{N_{R}\times N_{T}}$ given by

(1) $$\begin{array}{c} G\left(k\right)=\sqrt{\frac{1}{N_{c}}}\sum_{l=1}^{N_{c}}H_{l}e^{-j2\pi lk/N_{c}},\;\forall \;k\in \left\{1,\ldots ,N_{C}\right\} \end{array}$$

where Nc represents the number of sub-carriers over the whole bandwidth through an OFDM scenario, *k* is the index of the sub-carrier *k*, and $H_{l}\in C^{N_{R}\times N_{T}}$ is the narrow band channel model representing the time domain channel impulse response with L-tapped delays given by $H_{l}=\sqrt{\frac{N_{T}N_{R}}{L}}\sum_{i=1}^{L}\rho_{i}\;a_{R}\left(\theta_{i}^{\left(R\right)}\right)a_{T}^{H}\left(\theta_{i}^{\left(T\right)}\right)$, where *L* is number of paths (rank) of the channel; $\theta_{i}^{\left(R\right)}$ and $\theta_{i}^{\left(T\right)}$ are the angles of arrival at BS and the angles of departure from UE, noting AoA/AoD to correspond to the $i^{th}$ path (and both assumed to be uniform over [−π/2,π/2]); $\rho_{i}$ is the complex gain of the $i^{th}$ path such that $\rho_{i}∼\mathrm{CN}\left(0,1\right),\;\forall i$; and last but not least, $a_{R}\left(\theta_{i}^{\left(R\right)}\right)\in C^{N_{R}}$ and $a_{T}\left(\theta_{i}^{\left(T\right)}\right)\in C^{N_{T}}$ are the array response vectors at both UE and BS, respectively. We further assume that the channel is completely unknown to both UE and BS. Henceforth, in this paper, we shall denote the beam pair (u,i) as the combination of the UE beamformer indexed *u* from the UE codebook T and combiner indexed *i* in the BS codebook R. The signal at BS resulting from applying the beam pair (u,i), $y_{u,i}\in C^{N_{rf}}$ is expressed as

(2) $$\begin{array}{cc} y_{u,i}\; & ={W_{i}}^{H}G\left(k\right)\;f_{u}s_{u}+n_{i},\;\forall \;\left(u,i\right)\in T\times R, \end{array}$$

where $s_{u}=1\sqrt{P_{u}}$ is the transmitted pilot symbol associated with $f_{u}$ (having power $\sqrt{P_{u}}$) and $n_{i}=W_{i}^{H}n$ is the effective additive white Gaussian noise AWGN with unit variance ($\sigma^{2}=1$). We define the received Signal-to-Noise Ratio (SNR) for the beam pair (u,i) as ${\mathrm{SNR}}_{u,i}=P_{u}\left|\right|W_{i}^{H}G\left(k\right)f_{u}{\left|\right|}_{2}^{2},\;\forall \;\left(u,i\right)\in T\times R$. We assume a fully blind approach; i.e., neither BS nor UE has any knowledge of G. Thus, computing the above SNR expression is not feasible due to the fact that BS is assumed not to know G. Thus, in this work, we will approximate the SNR of the beam pair (u,i) using the corresponding instantaneous Received Signal Energies (RSEs) expressed as ${\mathrm{RSE}}_{u,i}=\left|\right|y_{u,i}{\left|\right|}_{2}^{2},\;\forall \left(u,i\right)\in T\times R$. In other words, we will assume that $RSE_{u,i}\approx {\mathrm{SNR}}_{u,i}$ for each beam pair (u,i)∈T×R.

**Figure 2:**
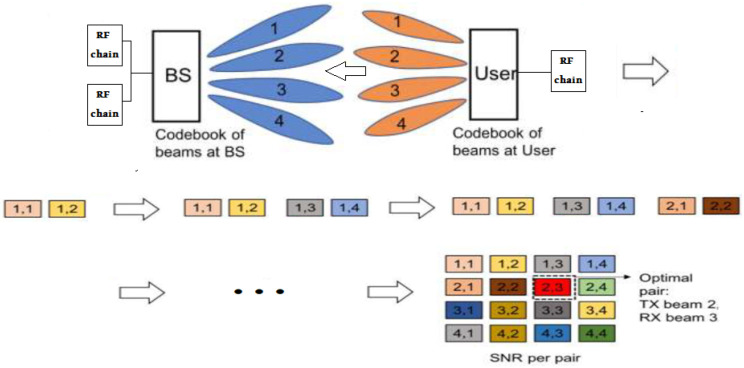
Exhaustive Beam Alignment: |T|=|R|=4, $N_{rf}=2$ RF-Chains at BS. Record 2 beam pairs for each pilot symbol transmission until the matrix is complete. Signaling overhead, $\Omega =\frac{4\times 4}{2}$.

**Benchmark: Exhaustive BA:** The de facto method for Beam Alignment is Exhaustive BA. It is accomplished by *exhaustively sounding*, jointly, the beams of both UE and BS codebooks, recording all entries of RSE, and exhaustively searching S for the indexes of the beam pair that maximize RSE at BS, i.e, $\left(u^{★},i^{★}\right)=\underset{(u,i)\in T\times R}{argmax}\;RSE_{u,i}$. Thus, the RSE matrix is computed/recorded $N_{rf}$-entries, with each of pilot symbol, since $N_{rf}$ samples are simultaneously received at the BS for every pilot transmission (see Figure 2). Consequently, the pilot signaling overhead of the Exhaustive BA is $\Omega =|T\times R|\;/\;N_{rf}=C_{T}\;C_{R}\;/\;N_{rf}$, which implies that the overhead of this benchmark scales poorly with the BS and UE codebooks.

**Proposed partial Beam Alignment using sub-sampled codebooks:** Recall the designation of the *beam pair* (u,i) as the beamforming vector of the index *u* in the UE codebook of beams and the combining vector of the index *i* in the BS codebook of beams. First, we select (at random) the indexes of the *sub-sampled codebooks* of beams at UE and BS, $R_{S}$ and $T_{S}$, such that $R_{S}\subset R$ and $T_{S}\subset T$, and $|R_{S}\left|\ll |R\right|$$|T_{S}\left|\ll |T\right|$. The idea behind this approach is to only sound beam pairs from the sub-sampled codebook of beams, $R_{S}$ and $T_{S}$. We thus define the *training set*, K, as the sub-sampled codebook indexes at UE and BS, i.e., $K:=\{\left(u,i\right)\;|\;\left(u,i\right)\in T_{S}\times R_{S}\}$. Then, the RSE of the sounded beam pairs (training set) is given to several ML methods, and the learned ML model is used to predict the RSE of non-sounded beam pairs.

We formalize this proposed method below. We express both the received signal $y_{\left(u,i\right)}$ and RSE for the beam pair (u,i) resulting from the sounded beam pairs (i.e., training set), as follows:

(3) $$\begin{array}{c} y_{u,i}={W_{i}}^{H}G\left(k\right)\;f_{u}s_{u}+n_{i},\forall \;\left(u,i\right)\in T_{S}\times R_{S} \end{array}$$

(4) $$\begin{array}{c} {\mathrm{RSE}}_{u,i}={∥y_{u,i}∥}_{2}^{2}\;,\forall \;\left(u,i\right)\in T_{S}\times R_{S}. \end{array}$$

**Figure 3:**
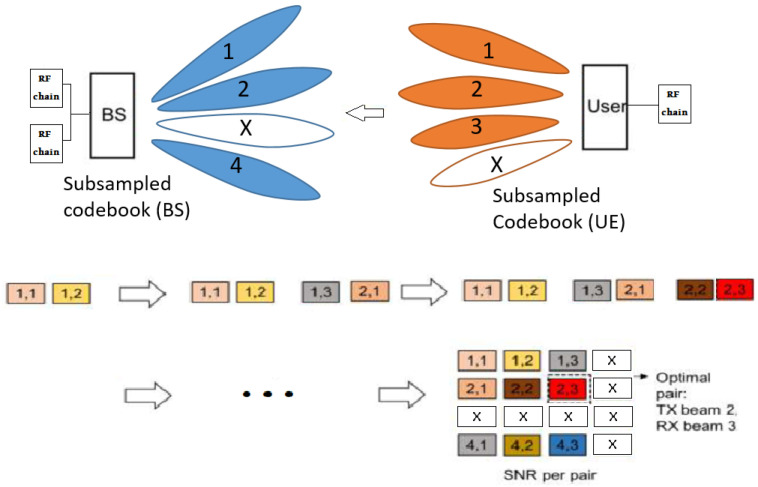
Proposed partial Beam Alignment using sub-sampled codebooks: |T|=|R|=4, $N_{rf}=2$ RF-Chains: record 2 beam pairs for each pilot symbol transmission until sounded beams are recorded. The missing entries represent the predicted entries. Signaling overhead, $\Omega =\frac{3\times 3}{2}$.

The dataset is formulated using the following incomplete RSE matrix, $S\in R^{C_{T}\times C_{R}}\left(:=R^{\left|T|\times |R\right|}\right)$:

(5) $$\begin{array}{c} {\left[S\right]}_{u,i}\;:=\left\{\begin{array}{cc} {\mathrm{RSE}}_{u,i}\; & ,if\;\left(u,i\right)\in T_{S}\times R_{S}\\ Unknown\;RSE\; & ,if\;\left(u,i\right)\notin T_{S}\times R_{S} \end{array}\right. \end{array}$$

where ${\left[S\right]}_{u,i}$ denotes the element (u,i) of S, ∀(u,i)∈T×R. Evidently, the value of RSE is undefined for the beam pairs that were not sounded, designated as unknown-RSE matrix coefficient. Those are the missing entries, which are predicted using one of the following proposed ML methods: (i) low-rank MF/NMF and (ii) shallow (feed-forward) MLP, where we utilize the sounded RSE entries as the *training set*, K. Then the training set, K, is fed into one of the above ML models, which will predict the RSE of non-sounded coefficients in S, denoted as ‘Unknown’, in (5) (see Figure 3). Finally, the pilot signaling overhead for the above-proposed sub-sampled codebook method is $\Omega =|T_{S}\times R_{S}|\;/\;N_{rf}=\left|K\right|\;/\;N_{rf}$. We split the RSE dataset into a training set K and a test set L such that K∩L={}. In this paper, $RSE_{u,i}$ denotes the true value (label) of the RSE for the beam pair (u,i) in the training set K, and  $\hat{RSE_{u,i}}$ denotes the true value (label) of the RSE for the beam pair (u,i) in the test set L.

**Signaling overhead ratio:** It is defined as $\eta :=\frac{overhead\;of\;learning-based\;BA}{overhead\;of\;Exhaustive\;BA}=\frac{|T_{S}\left|\times \right|R_{S}|}{\left|T|\times |R\right|}=\frac{\left|K\right|}{C_{T}C_{R}}$, where $T_{S}$ and $R_{S}$ are, respectively, the sizes of the UE and BS sub-sampled codebooks used in our proposed partial beam sounding, while T and R refer to the original size of the codebooks, and 0<η≤1 measures the signaling overhead of all the proposed MF, MLP, and AE methods compared with that of Exhaustive BA. Evidently, a small value for η is desired to reduce the signaling overhead of our proposed method. However, a low η implies that the size of the training set is small. As a result, the proposed ML method will not be able to extract enough data patterns due to the (too) small number of training samples, resulting in a larger prediction error. As one of the contributions of this work, we will (empirically) find as small a value for η as possible while still having extremely small training and prediction error.

***Conjecture:** Note that, from the equations of the narrow-band channel model H and the wide-band channel model G(k), it is simple to verify that rank(H)≤L and $rank\left(G\left(k\right)\right)\leq LN_{C}$. Assume that $P_{u}\to \infty$. Thus, we can approximate the RSE matrix as*

(6) $$\begin{array}{c} \;{\left[S\right]}_{u,i}=∥y_{u,i}{∥}_{2}^{2}=∥{W_{i}}^{H}G\left(k\right)\;f_{u}\sqrt{P_{u}}+n_{i}{∥}_{2}^{2}\overset{P_{u}\to \infty }{\approx }P_{u}{∥{W_{i}}^{H}G\left(k\right)\;f_{u}∥}_{2}^{2}\;,\;\forall \left(u,i\right)\in T\times R \end{array}$$

*If $P_{u}\to \infty$, then it can be shown that the RSE matrix S is such that $rank\left(S\right)\leq LN_{C}$. This implies that if $P_{u}\to \infty$, then $S\in R^{C_{R}\times C_{T}}$ is a low-rank matrix, i.e., $rank\left(S\right)\leq LN_{C}\ll \;\;\min\left(C_{T},C_{R}\right)$.*

While the proof for this necessary condition eludes the authors, we empirically observed that if $P_{u}$ is large, then the number of non-zero singular values of S, ${\left\{\sigma_{i}\left(S\right)\right\}}_{i=1}^{rank(S)}$, satisfies the above upper bound, i.e., $|\;{\left\{\sigma_{i}\left(S\right)\right\}}_{i=1}^{rank(S)}\;|\leq LN_{C}$.

**Remark 1.**  *Recall the expression for the effective rate, r, $r=\left(1-\frac{\Omega }{T}\right)\log\left(1+RSE_{u,i}\right)$, where* Ω *is the pilot signaling overhead and T is the number of symbols per block. Thus, the problem of maximizing r is written as the following series of equivalent problems:*

*$\left(u^{★},i^{★}\right):=\arg\max_{\forall (u,i)\in T\times R}r\Leftrightarrow \arg\max_{\forall (u,i)\in T\times R}\log\left(1+RSE_{u,i}\right)\Leftrightarrow \arg\max_{\forall (u,i)\in T\times R}$$RSE_{u,i}$, where the last* ⇔ *is due to the fact that the log(x) is a strictly monotonically increasing function in x. This result implies finding the optimal beam pair $\left(u^{★},i^{★}\right)$ that maximizes r is equivalent to finding the best beam pair that maximizes the RSE.*

**Remark 2.**  *The information (number of entries) needed to represent the RSE matrix $S\in C^{C_{R}\times C_{T}}$ is measured as $rank\left(S\right)\left(1+C_{T}+C_{R}\right)$. This result is evident from performing the SVD on S and counting the resulting number of entries. Thus, if S is severely rank deficient, i.e., extremely compressible, then methods such as MF/NMF will exhibit extremely small training and test error. Conversely, if S is full rank, i.e., not compressible, then the training and test of MF/NMF will be quite large.*

## 4. Matrix Factorization and Non-Negative Matrix Factorization

### 4.1. MF and NMF Problem Formulation

**Figure 4:**
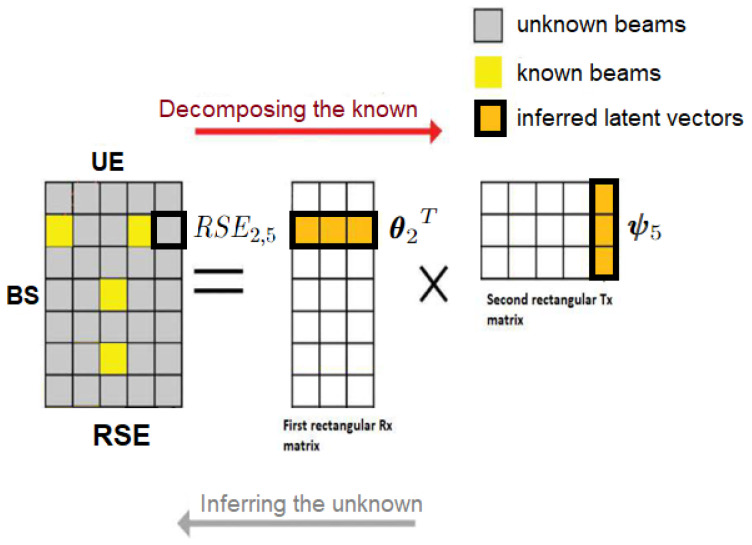
Toy Example: Matrix Factorization with |T|=5,|R|=7,D=3. MF results into two rectangular matrices to be optimized: MF uses the RSE of known beams (yellow) to predict/complete unknown beams (gray). The product of the latent factors $\theta_{2}^{T}$ and $\psi_{5}$ gives the unknown value of $RSE_{2,5}$.

The intuition behind low-rank MF is to model the RSE of the sounded beam pairs (i.e., entries of S that are known as $T_{S}\times R_{S}$) as an inner product between two *D*-dimensional latent vectors/factors, $\theta_{u},\psi_{i}$, as illustrated in Figure 4. Specifically, the RSE of the beam pair (u,i), denoted as ${\left[S\right]}_{u,i}$, is modeled as ${\left[S\right]}_{u,i}:=\theta_{u}^{T}\psi_{i}\;,\theta_{u}\in R^{D}\;,\;\psi_{i}\;\in \;R^{D}$, $\forall \;\left(u,i\right)\in K\left(:=T_{S}\times R_{S}\right)$, where *D* is the size/dimension/complexity of the Matrix Factorization model latent factors and $\theta_{u}\in R^{D}\;,\;\psi_{i}\;\in \;R^{D}$ are the MF model parameters (to be optimized). In addition, due to the low-rank MF model, *D* is assumed to be much smaller than the dimensions of S, i.e., $D\ll (C_{T},C_{R})$. The RSE of the beam pair (u,i) is known from sounding the sub-sampled codebooks (i.e., label). The general formulation of our loss function $\ell_{u,i}$ describes the distance between the true value $RSE_{u,i}$ and the predicted value $\theta_{u}^{T}\psi_{i}$, which corresponds to the MF output/prediction: $\ell_{u,i}:={\left(RSE_{u,i}-\theta_{u}^{T}\psi_{i}\right)}^{2}\;,\forall \;\left(u,i\right)\in K\left(:=T_{S}\times R_{S}\right)$. The Empirical Risk (also known as training error) is defined as the average across all the individual loss function $\ell_{u,i}$. We define the regularized Empirical Risk function as the above empirical risk in addition to the following regularization terms:

(7) $$\begin{array}{c} \sum_{(u,i)\in K}\;\left[\frac{1}{\left|K\right|}{\left({\left[S\right]}_{u,i}-\theta_{u}^{T}\psi_{i}\;\right)}^{2}+\lambda_{i}∥\psi_{i}{∥}_{2}^{2}+\mu_{u}{∥\theta_{u}∥}_{2}^{2}\right]=f\left({\left(\theta_{u},\psi_{i}\right)}_{(u,i)\in K}\right) \end{array}$$

where $\left\{\lambda_{i}\geq 0,\mu_{u}\geq 0\;|\;\forall \left(u,i\right)\in K\right\}$ is the set of regularization hyperparameters used to balance the MF/NMF model, preventing any overfitting or underfitting. The Empirical Risk Minimization corresponding to the MF model is given by

$$\begin{array}{c} \left(P1\right):=\left\{{\hat{\theta }}_{u},{\hat{\psi }}_{i}\right\}\;\;\left\{\begin{array}{c} \underset{{\left\{\theta_{u},\psi_{i}\right\}}_{(u,i)\in K}}{argmin}\;f\left(\theta_{u},\psi_{i}\right)\\ s.t.\;\theta_{u}\;\in \;R^{D},\;\psi_{i}\;\in \;R^{D} \end{array}\right. \end{array}$$

For the Matrix Factorization variant NMF, the optimization problem is given by

$$\begin{array}{c} \left(P2\right):=\left\{{\hat{\theta }}_{u},{\hat{\psi }}_{i}\right\}\;\;\left\{\begin{array}{c} \underset{{\left\{\theta_{u},\psi_{i}\right\}}_{(u,i)\in K}}{argmin}\;f\left(\theta_{u},\psi_{i}\right)\\ s.t.\;\theta_{u}\;\in \;R_{+}^{D},\;\psi_{i}\;\in \;R_{+}^{D} \end{array}\right. \end{array}$$

where $\left\{{\hat{\theta }}_{u},{\hat{\psi }}_{i}\right\}$ denotes the optimal latent vectors for MF and NMF. The test loss (also knows as test error) is given by applying the general loss on the unknown data samples (non-sounded beams) using optimal MF/NMF parameters ${\hat{\theta }}_{u}$ and ${\hat{\psi }}_{i}$: $=\frac{1}{\left|L\right|}\sum_{(u,i)\in L}\;{\left(\;\hat{RSE_{u,i}}-{\hat{\theta }}_{u}^{T}{\hat{\psi }}_{i}\;\right)}^{2}$, where L is the test set of our learning model.

### 4.2. Solutions for MF

We resolve the MF problem (P1) using the following methods: (i) Block Coordinate Descent (BCD) often denoted as Alternating Least Squares (ALSs), (ii) BCD with Stochastic Gradient Descent, and (iii) Block Gradient Descent (BGD), which merges BCD and Gradient Descent (GD) definitions.

**BCD for MF (BCD MF):** BCD proceeds by splitting the optimizing problem (P1) into sub-problems, supposing that all other blocks are known/fixed. We will show that each sub-problem is strongly convex in each block, and the BCD algorithm converges to a stationary point. The application of BCD to the MF problem results in two sub-problems, S1 and S2, which are solved iteratively. At iteration *k*, the sub-problem (S1) is defined by fixing the block ${\left\{\psi_{i}^{\left(k\right)}\right\}}_{\forall i}$ and the update/solve block ${\left\{\theta_{u}\right\}}_{\forall u}$ only, as follows:

$$\begin{array}{cc} \left(S1\right):\;\theta_{u}^{\left(k+1\right)}\; & =argmin_{\theta_{u}\in R^{d}}\;f\left(\left\{\theta_{u}\;,\psi_{i}^{\left(k\right)}\right\}\right)\\ \; & =\sum_{(u,i)\in K}\;[{\left({\left[S\right]}_{u,i}-\theta_{u}^{T}\psi_{i}^{\left(k\right)}\right)}^{2}+\mu_{u}∥\theta_{u}{∥}_{2}^{2}+\lambda_{i}∥\psi_{i}^{\left(k\right)}{∥}_{2}^{2}] \end{array}$$

Moreover, the sub-problem (S2) is defined by fixing the block ${\left\{\theta_{u}^{\left(k+1\right)}\right\}}_{\forall u}$ in $\left(P_{1}\right)$ and the update/solve block ${\left\{\psi_{i}\right\}}_{\forall i}$, only, as follows:

$$\begin{array}{cc} \left(S2\right):\;\psi_{i}^{\left(k+1\right)}\; & =argmin_{\theta_{i}\in R^{d}}\;f\left(\left\{\theta_{u}^{\left(k+1\right)}\;,\psi_{i}\right\}\right)\\ \; & =\sum_{(u,i)\in K}\;[{\left({\left[S\right]}_{u,i}-\theta_{u}^{\left(k+1\right)}\psi_{i}\right)}^{2}+\mu_{u}∥\theta_{u}^{\left(k+1\right)}{∥}_{2}^{2}+\lambda_{i}∥\psi_{i}{∥}_{2}^{2}] \end{array}$$

We will rewrite S1 into as series of equivalent problems as follows:

$$\begin{array}{cc} (S1)\; & :=argmin_{\theta_{u}\in R^{d}}\;\sum_{(u,i)\in K}\;[{\left[S\right]}_{u,i}^{2}-2{\left[S\right]}_{u,i}\theta_{u}^{T}\psi_{i}^{\left(k\right)}+\theta_{u}^{T}\psi_{i}^{\left(k\right)}\theta_{i}^{{\left(k\right)}^{T}}\theta_{u}+\mu_{u}∥\theta_{u}{∥}_{2}^{2}]\\ & \Leftrightarrow argmin_{\theta_{u}\in R^{d}}\;\sum_{u}\;[-2\theta_{u}^{T}\;\sum_{i}\left({\left[S\right]}_{u,i}\psi_{i}^{\left(k\right)}\right)+\theta_{u}^{T}\;\sum_{i}\left(\psi_{i}^{\left(k\right)}\psi_{i}^{{\left(k\right)}^{T}}\right)\theta_{u}+\mu_{u}∥\theta_{u}{∥}_{2}^{2}]\\ & \Leftrightarrow argmin_{\theta_{u}\in R^{d}}\;\sum_{u\in U_{i}}\;[-2\theta_{u}^{T}\;\left(r_{u}^{\left(k\right)}\right)+\theta_{u}^{T}\left(Q_{u}^{\left(k\right)}\right)\theta_{u}+\mu_{u}∥\theta_{u}{∥}_{2}^{2}]=\sum_{u\in U_{i}}h_{u}\left(\theta_{u}\right),\\ \theta_{u}^{\left(k+1\right)} & =argmin_{\theta_{u}\in R^{d}}\;\;\left[-2\theta_{u}^{T}r_{u}^{\left(k\right)}+\theta_{u}^{T}\left(\;Q_{u}^{\left(k\right)}+\mu_{u}I_{D}\;\right)\theta_{u}\right]\;=\;f_{1}\left(\theta_{u}\right),\;\;\forall u\in U_{i}, \end{array}$$

where $U_{i}$ is the set of row indexes *u* in the RSE matrix corresponding to the column *i* in the known entries of the RSE matrix, $Q_{u}^{\left(k\right)}=\sum_{i}\left(\psi_{i}^{\left(k\right)}\psi_{i}^{{\left(k\right)}^{T}}\right)$ and $r_{u}^{\left(k\right)}=\sum_{i}\left({\left[S\right]}_{u,i}\psi_{i}^{\left(k\right)}\right)$. We derive the closed-form solution for the sub-problem S1 by finding the global min of $f_{1}\left(\theta_{u}\right)$, as follows:

$$\begin{array}{cc} \nabla f_{1}\left(\theta_{u}\right)=0 & \Leftrightarrow -2r_{u}^{\left(k\right)}+2\left(\;Q_{u}^{\left(k\right)}+\mu_{u}I_{D}\;\right)\theta_{u}=0\Leftrightarrow \theta_{u}={\left(\;Q_{u}^{\left(k\right)}+\mu_{u}I_{D}\;\right)}^{-1}r_{u}^{\left(k\right)} \end{array}$$

Similarly, we rewrite the sub-problem (S2) into the following series of equivalent problems by stating the last one:

$$\begin{array}{cc} \left(S2\right):\psi_{i}^{\left(k+1\right)}= & argmin_{\psi_{i}\in R^{d}}\;\left[-2t_{i}^{{\left(k+1\right)}^{T}}\psi_{i}+{\psi_{i}}^{T}\left(\;P_{i}^{\left(k+1\right)}+\lambda_{i}I\;\right)\psi_{i}\right]=f_{2}\left(\psi_{i}\right)\;,\;\forall \;i\in I_{u}, \end{array}$$

where $I_{u}$ is the set of column indexes *i* in the RSE matrix corresponding to the row *u* in the known entries of the RSE matrix, $t_{i}^{\left(k+1\right)}=\sum_{u}\left(\;{\left[S\right]}_{u,i}\theta_{u}^{{\left(k+1\right)}^{T}}\right)$ and $P_{i}^{\left(k+1\right)}=\sum_{u}\left(\;\theta_{u}^{\left(k+1\right)}\theta_{u}^{{\left(k+1\right)}^{T}}\;\right)$. Next, we derive a closed-form solution for the sub-problem S2 by finding the global min of $f_{2}\left(\psi_{i}\right)$, as follows:

$$\begin{array}{c} \nabla f_{2}\left(\psi_{i}\right)=0\Leftrightarrow -2t_{i}^{\left(k+1\right)}+2\left(\;P_{i}^{\left(k+1\right)}+\lambda_{i}I_{D}\;\right)\psi_{i}=0\Leftrightarrow \psi_{i}={\left(\;P_{i}^{\left(k+1\right)}+\lambda_{i}I_{D}\;\right)}^{-1}t_{i}^{\left(k+1\right)}\\ ↔\psi_{i}^{\left(k+1\right)}={\left(\left(\sum_{u}\left(\;\theta_{u}^{\left(k+1\right)}\theta_{u}^{{\left(k+1\right)}^{T}}\;\right)\right)+\lambda_{i}I_{D}\;\right)}^{-1}\left(\sum_{u}\left(\;{\left[S\right]}_{u,i}\theta_{u}^{{\left(k+1\right)}^{T}}\right)\right) \end{array}$$

Thus, BCD updates to solve MF are given as follows:

$$\begin{array}{c} \left\{\begin{array}{cc} \theta_{u}^{\left(k+1\right)} & =\left(\sum_{i}\psi_{i}^{\left(k\right)}{\left(\psi_{i}^{\left(k\right)}\right)}^{T}\right)+\mu_{u}{I)}^{-1}\left(\sum_{i}{\left[S\right]}_{u,i}\psi_{i}^{\left(k\right)}\right)\\ \psi_{i}^{\left(k+1\right)} & ={\left(\left(\sum_{u}\theta_{u}^{\left(k+1\right)}{\left(\theta_{u}^{\left(k+1\right)}\right)}^{T}\right)+\lambda_{i}I\right)}^{-1}\left(\sum_{u}{\left[S\right]}_{u,i}\theta_{u}^{\left(k+1\right)}\right) \end{array}\right. \end{array}$$

(8) $$\forall \left(u,i\right)\in K\;,\;k=0,1,\ldots ,I_{M}\;$$

where ^(*k*)^ represents the index of the BCD iterations, (*u*,*i*) are the codebook indexes at UE and BS, and ${\left[S\right]}_{u,i}$ denotes the RSE of the (*u*,*i*) beam couple. The solution ${\left\{{\hat{\theta }}_{u},{\hat{\psi }}_{i}\right\}}_{(u,i)\in K}$ is reached after the interval/gap between consecutive iterations reaches a predefined ϵ or a max number of iterations, $I_{M}$. We have the following result.

**Corollary 1.**  *The sequence of updates ${\left\{\theta_{u}^{\left(k\right)},\psi_{i}^{\left(k\right)}\;|\;\forall \left(u,i\right)\in K\right\}}_{k}$ generated by BCD, in* (8)*, is non-increasing (in k) and converges to a stationary point as k→∞.*

**Proof.**  See Appendix A. □

**Block Stochastic Gradient Descent (BSGD) for MF (SGD MF):** SGD MF proceeds by taking *T* plain SGD steps (mini-batch size =1). BGD proceeds by taking *T* SGD steps for each block BCD. We first choose at random a single training sample (u,i)∈K. The BSGD update for the sub-problem (S1) is done by performing SGD for $f_{1}\left(\theta_{u}\right)=\sum_{u\in U_{i}}h_{u}\left(\theta_{u}\right)$, i.e., choosing at random a single index $u\in U_{i}$ and computing the plain SGD $\hat{\nabla f_{1}\left(\theta_{u}\right)}=\hat{\nabla }\left(\sum_{u\in U_{i}}h_{u}\left(\theta_{u}\right)\right)=h_{u}\left(\theta_{u}\right)$, where *u* is a random index from $U_{i}$, and $\hat{\nabla f_{1}\left(\theta_{u}\right)}$ is the plain SGD on $f_{1}\left(\right)$. The corresponding update is given as

$$\begin{array}{cc} \theta_{u}^{\left(k+1\right)} & =\theta_{u}^{\left(k\right)}-\alpha_{k}\hat{\nabla f_{1}\left(\theta_{u}^{\left(k\right)}\right)}\;,=\theta_{u}^{\left(k\right)}-\alpha_{k}\nabla h_{u}\left(\theta_{u}^{\left(k\right)}\right)\;u\;\in U_{i}\\ \; & =\theta_{u}^{\left(k\right)}+2\alpha_{k}\left(\;\left(\sum_{i}\left({\left[S\right]}_{u,i}\psi_{i}^{\left(k\right)}\right)\right)-\left(\;\left(\sum_{i}\psi_{i}^{\left(k\right)}\psi_{i}^{{\left(k\right)}^{T}}\right)+\mu_{u}I_{D}\;\right)\theta_{u}^{\left(k\right)}\right),\;u\;\in U_{i},\;k=1..T \end{array}$$

where *u* is a single index chosen at random from $U_{i}$, $Q_{u}^{\left(k\right)}=\sum_{i}\left(\psi_{i}^{\left(k\right)}\psi_{i}^{{\left(k\right)}^{T}}\right)$, $r_{u}^{\left(k\right)}=\sum_{i}\left({\left[S\right]}_{u,i}\psi_{i}^{\left(k\right)}\right)$, ^(*k*)^ is the iteration index for SGD, and $\hat{\nabla f_{1}\left(\theta_{u}\right)}$ is the plain SGD over one random sample $u\in U_{i}$. Similarly, the update for the sub-problem (S2) is done by taking *T* plain SGD steps of $f_{2}\left(\psi \right)=\sum_{i\in I_{u}}h_{i}\left(\psi_{i}\right)$, i.e., the SGD, $\hat{\nabla f_{2}\left(\psi_{i}\right)}=\hat{\nabla }\left(\sum_{i\in I_{u}}h_{i}\left(\psi_{i}\right)\right)=h_{i}\left(\psi_{i}\right)$, where *i* is single random index from $I_{u}$. Thus, the SGD MF update for the sub-problem (S2) is expressed as

$$\begin{array}{cc} \psi_{i}^{\left(k+1\right)} & =\psi_{i}^{\left(k\right)}-\alpha_{k}\hat{\nabla f_{2}\left(\psi_{i}^{\left(k\right)}\right)}=\psi_{i}^{\left(k\right)}-\alpha_{k}\nabla h_{2}\left(\psi_{i}^{\left(k\right)}\right)\;,\;i\in I_{u}\\ \; & =\psi_{i}^{\left(k\right)}+2\alpha_{k}\left(\;\left(\sum_{u}\left(\;{\left[S\right]}_{u,i}\theta_{u}^{{\left(k\right)}^{T}}\right)\right)-\left(\;\sum_{u}\left(\;\theta_{u}^{\left(k\right)}\theta_{u}^{{\left(k\right)}^{T}}\;\right)\;\right)+\lambda_{i}I_{D}\;\right)\theta_{u}^{\left(k\right)}),i\in I_{u},\;\forall k=1..T \end{array}$$

where *i* is a single index chosen randomly from $I_{u}$, $t_{i}^{\left(k\right)}=\sum_{u}\left(\;{\left[S\right]}_{u,i}\theta_{u}^{{\left(k\right)}^{T}}\right)$, $P_{i}^{\left(k\right)}=\sum_{u}\left(\;\theta_{u}^{\left(k\right)}\theta_{u}^{{\left(k\right)}^{T}}\;\right)$, and $\hat{\nabla f_{2}\left(\psi_{i}\right)}$ is the plain SGD gradient computed with one sample $i\in I_{u}$, chosen at random. We write the SGD MF updates as

$$\begin{array}{c} \left\{\begin{array}{cc} \theta_{u}^{\left(k+1\right)} & =\theta_{u}^{\left(k\right)}+2\alpha_{k}\left(\;\left(\sum_{i}\left({\left[S\right]}_{u,i}\psi_{i}^{\left(k\right)}\right)\right)-\left(\;\left(\sum_{i}\psi_{i}^{\left(k\right)}\psi_{i}^{{\left(k\right)}^{T}}\right)+\mu_{u}I_{D}\;\right)\theta_{u}^{\left(k\right)}\right),\;u\in U_{i}\\ \psi_{i}^{\left(k+1\right)} & =\psi_{i}^{\left(k\right)}+2\alpha_{k}\left(\;\left(\sum_{u}\left(\;{\left[S\right]}_{u,i}\theta_{u}^{{\left(k\right)}^{T}}\right)\right)-\left(\;\sum_{u}\left(\;\theta_{u}^{\left(k\right)}\theta_{u}^{{\left(k\right)}^{T}}\;\right)\;\right)+\lambda_{i}I_{D}\;\right)\theta_{u}^{\left(k\right)}),\;i\in I_{u} \end{array}\right. \end{array}$$

(9) ∀k=0,1,…,T,

where *u* is a random index chosen from $U_{i}$, and *i* a random index from $I_{u}$. $0\leq \alpha_{k}\leq 1$ is the step size for SGD.

**BGD for MF (BGD MF):** Rather than having a closed-form solution for each optimization block, BGD proceeds by taking *T* gradient steps for each block *T* gradient step. We skip the details here for space limitations. Thus, the BGD updates for the MF problem are expressed as

$$\begin{array}{c} \left\{\begin{array}{cc} \theta_{u}^{\left(k+1\right)} & =\theta_{u}^{\left(k\right)}+2\alpha_{k}\left(\;\left(\sum_{i}\left({\left[S\right]}_{u,i}\psi_{i}^{\left(k\right)}\right)\right)-\left(\;\left(\sum_{i}\psi_{i}^{\left(k\right)}\psi_{i}^{{\left(k\right)}^{T}}\right)+\mu_{u}I_{D}\;\right)\theta_{u}^{\left(k\right)}\right)\\ \psi_{i}^{\left(k+1\right)} & =\psi_{i}^{\left(k\right)}+2\alpha_{k}\left(\;\left(\sum_{u}\left(\;{\left[S\right]}_{u,i}\theta_{u}^{{\left(k\right)}^{T}}\right)\right)-\left(\;\sum_{u}\left(\;\theta_{u}^{\left(k\right)}\theta_{u}^{{\left(k\right)}^{T}}\;\right)\;\right)+\lambda_{i}I_{D}\;\right)\theta_{u}^{\left(k\right)}) \end{array}\right. \end{array}$$

(10) ∀(u,i)∈K,k=0,1,…,T,

where (*u*,*i*) are the codebook indexes at UE and BS, *k* is the GD iteration index, and $\alpha^{\left(k\right)}$ is the BGD step size ($0<\alpha^{\left(k\right)}<1$).

### 4.3. Solutions for NMF

Our proposed NMF follows the exact steps as in MF, with the main difference of constraining the latent vectors being non-negative $\theta_{u}\in R_{+}^{D}\;,\;\psi_{i}\in R_{+}^{D},\forall \;\left(u,i\right)\in K$. Likewise, we solve the NMF problem, (P2), using BCD, SGD, and BGD.

**BCD for NMF (BCD NMF):** The derivations of BCD for NMF (11) are identical to those of BCD for MF (8), followed by the corresponding projection operation. The updates of BCD for NMF derivations are given by

$$\begin{array}{c} \left\{\begin{array}{cc} \theta_{u}^{\left(k+1\right)} & ={\left[\left(\sum_{i}\psi_{i}^{\left(k\right)}{\left(\psi_{i}^{\left(k\right)}\right)}^{T}\right)+\mu_{u}{I)}^{-1}\left(\sum_{i}{\left[S\right]}_{u,i}\psi_{i}^{\left(k\right)}\right)\right]}_{+}\\ \psi_{i}^{\left(k+1\right)} & ={\left[{\left(\left(\sum_{u}\theta_{u}^{\left(k+1\right)}{\left(\theta_{u}^{\left(k+1\right)}\right)}^{T}\right)+\lambda_{i}I\right)}^{-1}\left(\sum_{u}{\left[S\right]}_{u,i}\theta_{u}^{\left(k+1\right)}\right)\right]}_{+} \end{array}\right. \end{array}$$

(11) $$\forall \left(u,i\right)\in K\;,\;k=0,1,\ldots ,I_{M}\;$$

where ^(*k*)^ is the BCD iteration index, and ${\left[a\right]}_{+}:=\max\left(a,0\right)$ is applied element by element on a, i.e., a Euclidean projection of a on $R_{+}^{D}$. Since the projection is Euclidean (non-expansive operator), the corollary stated in the previous subsection applies to the BCD for NMF too.

**Block Stochastic Gradient Descent (BSGD) for NMF (SGD NMF):** The SGD NMF derivations are exactly the same as that of SGD MF, followed by a projection ${\left[\right]}_{+}$. We thus express the SGD NMF updates as

$$\begin{array}{c} \left\{\begin{array}{cc} \theta_{u}^{\left(k+1\right)} & ={\left[\theta_{u}^{\left(k\right)}+2\alpha_{k}\left(\;\left(\sum_{i}\left({\left[S\right]}_{u,i}\psi_{i}^{\left(k\right)}\right)\right)-\left(\;\left(\sum_{i}\psi_{i}^{\left(k\right)}\psi_{i}^{{\left(k\right)}^{T}}\right)+\mu_{u}I_{D}\;\right)\theta_{u}^{\left(k\right)}\right)\right]}_{+},\;u\in U_{i}\\ \psi_{i}^{\left(k+1\right)} & ={\left[\psi_{i}^{\left(k\right)}+2\alpha_{k}\left(\;\left(\sum_{u}\left(\;{\left[S\right]}_{u,i}\theta_{u}^{{\left(k\right)}^{T}}\right)\right)-\left(\;\sum_{u}\left(\;\theta_{u}^{\left(k\right)}\theta_{u}^{{\left(k\right)}^{T}}\;\right)\;\right)+\lambda_{i}I_{D}\;\right)\theta_{u}^{\left(k\right)})\right]}_{+},\;i\in I_{u} \end{array}\right. \end{array}$$

(12) ∀k=0,1,…,T,

where *u* is a random index chosen from $U_{i}$, *i* is a random index from $I_{u}$, ${\left[a\right]}_{+}:=\max\left(a,0\right)$, and $\alpha^{\left(k\right)}$ is the SGD step size ($0<\alpha^{\left(k\right)}<1$).

**BGD for NMF (BGD NMF):** The solution and derivations for BGD NMF are the same as those for BGD MF, followed by a projection ${\left[\right]}_{+}$, i.e,

$$\begin{array}{c} \left\{\begin{array}{cc} \theta_{u}^{\left(k+1\right)} & ={\left[\theta_{u}^{\left(k\right)}+2\alpha_{k}\left(\;\left(\sum_{i}\left({\left[S\right]}_{u,i}\psi_{i}^{\left(k\right)}\right)\right)-\left(\;\left(\sum_{i}\psi_{i}^{\left(k\right)}\psi_{i}^{{\left(k\right)}^{T}}\right)+\mu_{u}I_{D}\;\right)\theta_{u}^{\left(k\right)}\right)\right]}_{+}\\ \psi_{i}^{\left(k+1\right)} & ={\left[\psi_{i}^{\left(k\right)}+2\alpha_{k}\left(\;\left(\sum_{u}\left(\;{\left[S\right]}_{u,i}\theta_{u}^{{\left(k\right)}^{T}}\right)\right)-\left(\;\sum_{u}\left(\;\theta_{u}^{\left(k\right)}\theta_{u}^{{\left(k\right)}^{T}}\;\right)\;\right)+\lambda_{i}I_{D}\;\right)\theta_{u}^{\left(k\right)})\right]}_{+} \end{array}\right. \end{array}$$

(13) ∀(u,i)∈K,k=0,1,…,T,

where ${\left[a\right]}_{+}:=\max\left(a,0\right)$, ^(*k*)^ is the GD iteration index and $\alpha^{\left(k\right)}$ is the GD step size ($0<\alpha^{\left(k\right)}<1$). We use a constant step size $\alpha_{k}=\alpha$ for all these methods.

### 4.4. Prediction for MF and NMF

For both MF and NMF, the predicted RSE of the beam-pair (u,i), for beam indexes that were not sounded, is expressed as

(14) $$\begin{array}{c} \left\{{\hat{RSE}}_{u,i}:={\left({\hat{\theta }}_{u}\right)}^{T}{\hat{\psi }}_{i}\;|\;\forall \left(u,i\right)\in L\right\} \end{array}$$

where L is the test set and ${\left\{{\hat{\theta }}_{u}\right)}^{T},{\hat{\psi }}_{i}\}$ are optimal solutions to MF (or NMF). Afterwards, we search for the optimal beam pair at UE and BS as the one with the highest RSE value over both training and test sets, as follows:

(15) $$\begin{array}{c} \left(u^{★},i^{★}\right)=argmax_{(u,i)\in L\cup K}\;\;{\left({\hat{\theta }}_{u}\right)}^{T}{\hat{\psi }}_{i}. \end{array}$$

### 4.5. Proposed BA Algorithm Using MF/NMF

Due to the fact that the updates given in a closed-form solution, we can quantify the computational complexity of all of the above methods. As seen from the updates for BCD MF and BCD NMF, we have to invert two D×D matrices (for the sum problems S1 and S2). Thus, the (per-iteration) computational complexity of BCD MF and BCD NMF is approximated as $C_{BCD\;MF}=C_{BCD\;NMF}=O\left(2D^{3}\right)$. Moreover, for BGD MF and BGD NMF, one has to compute two full-batch gradients over all training samples in K (for the sub-problems S1 and S2). Consequently, the complexity, per-iteration, for BGD MF and BGD NMF is approximated as $C_{BGD\;MF}=C_{BGD\;NMF}=O\left(2|K|\right)$. Finally, for SGD MF and SGD NMF, since we use a mini-batch size =1 (for the sub-problems S1 and S2), the resulting per-iteration computational complexity is approximated as $C_{SGD\;MF}=C_{SGD\;NMF}=O\left(2\right)$. Solving the MF and NMF problem, we employ methods such as BCD, BGD, or SGD. All details are shown in Algorithm 1.
**Algorithm 1** Proposed MF/NMF-Based BA Method.Input: ${\left\{f_{u}\right\}}_{\forall u\in T}$, ${\left\{W_{i}\right\}}_{\forall i\in R}$, η, Pu-Generate randomly sub-sampled codebooks, $T_{S},R_{S}$, satisfying $\left(\right|T_{S}\left|.\right|R_{S}\left|\right)/\left(|T\right|\times \left|R|\right)=\eta$-Sound beam pairs from training set, $K:=T_{S}\times R_{S}$.-Record corresponding RSE in and generate mat. S, in (5)-Select model: MF or NMF-**IF** MF model selected
solve (P1) with BCD for MF, in (8) or solve (P1) with BGD for MF, in (10) or solve (P1) with SGD for MF, in (9). At the end of training, return optimal latent vectors, ${\left\{{\hat{\theta }}_{u},{\hat{\psi }}_{i}\right\}}_{(u,i)\in K}$
-**IF** NMF model selected
solve (P2) with BCD for NMF, in (11) or solve (P2) with BGD for NMF, in (13) or solve (P2) with SGD for NMF, in (12). At the end of training, return ideal latent vectors, ${\left\{{\hat{\theta }}_{u},{\hat{\psi }}_{i}\right\}}_{(u,i)\in K}$
-Use ideal latent vectors ${\left\{{\hat{\theta }}_{u},{\hat{\psi }}_{i}\right\}}_{(u,i)\in K}$, to predict unknown RSE of test set, L, in (14)-Search training and test sets, for beam pair w/ largest RSE, (15)Output: $f_{u^{★}}$, $W_{i^{★}}$

While, for MF BCD and NMF BCD, the only hyperparameter is the model size *D*, MF BGD and NMF BGD require, in addition to *D*, $\alpha^{k}$, the GD step size as hyperparameters.

### 4.6. Numerical Simulations

This section illustrates our numerical setup. The number of antennas at UE and BS∈{128, 256, 512, 1024}. We set up $N_{T}=C_{T}$ and $N_{R}=C_{R}$. The overhead ratio regime η∈{0.7,0.5,0.3,0.1}. The number of OFDM sub-carriers $N_{c}=64$ and the number of channel paths *L* is 2. We vary the transmitted power, $P_{u}\in \left\{1,10^{-1},10^{-2}\right\}$. We use DFT codebooks at UE and BS. The optimal hyperparameters are chosen to minimize test loss. The model dimension D∈{2,3,4,5,6}, the learning rate $\alpha_{k}\in \left\{10^{-1},10^{-2},10^{-3},10^{-4},10^{-5},10^{-6}\right\}$, and the regularization factors $\left\{\lambda ,\mu \right\}\in \left\{10^{-2},10^{-3},10^{-4},10^{-5},10^{-6},10^{-7}\right\}$. For each MIMO configuration and for each $P_{u}$ regime, we randomly generate and store the resulting RSE matrices.

**Figure 5:**
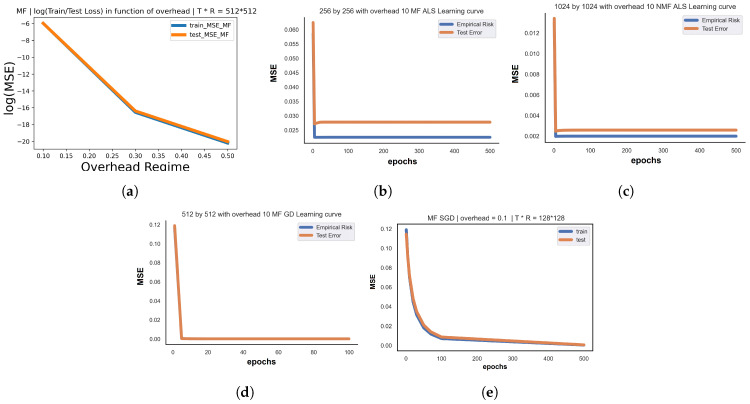
MF/NMF train/test performance and learning curves: (**a**) 512 × 512 train/test loss in function of the overhead ratio; (**b**) learning curve: 256 × 256 with overhead 0.1 BCDMF; (**c**) learning curve: 1024 × 1024 with overhead 0.1 BCDNMF; (**d**) learning curve: 512 × 512 with overhead 0.1 BGDMF; (**e**) learning curve: 128 × 128 with overhead 0.1 BCDSGD.

We propose to investigating six models in total (BCD MF, BCD NMF, BGD MF, BGD NMF, SGD MF, SGD NMF) with respect to three transmitted power regimes: high Pu=1W, medium $Pu=10^{-1}$ W, and low $Pu=10^{-2}$ W with fixed $\sigma^{2}=1$. In Table 1, we provide a summary for all proposed system parameters. We use the training Normalized MSE (NMSE) to evaluate the training error, expressed as $Train\;NMSE=\frac{1}{\left|K\right|}\left(\sum_{(u,i)\in K}{\left(\frac{\hat{\theta_{u}^{T}}\hat{\psi_{i}}-RSE_{u,i}}{RSE_{u,i}}\right)}^{2}\right)$. We also define $Test\;NMSE=\frac{1}{\left|L\right|}\left(\sum_{(u,i)\in L}{\left(\frac{\hat{RSE_{u,i}}-\hat{\theta_{u}^{T}}\hat{\psi_{i}}}{\hat{RSE_{u,i}}}\right)}^{2}\right)$. The range of training error and the overall behavior of BCD-based models are different and distinctive from GD models in both MF and NMF; for instance, BGD-based models’ error range are around $\times 10^{-7}$, while BCD-based models are around $\times 10^{-4}$. Thus, GD is more accurate. However, BCD converges faster and the cost function drops to low values from the very first iterations. In addition, for MF and NMF, the train NMSE decreases with the increase in the overhead ratio η, as seen in Figure 5. Low and medium $P_{u}$ regimes are characterized by noisy links between UE and BS and represent a more challenging experimental environment. BCD-based models tend to be faster in reaching low error values, while BGD-based models are more accurate. (For instance, BSGD generally ameliorates the quality of prediction compared with BGD).

Regarding MF/NMF simulation figures, Figure 5a states the decrease of train/test NMSE in function of the overhead ratio (more training samples result in fewer errors); Figure 5b,c track the instant drop in loss values from the very first iterations for BCD-based models; and Figure 5d,e present the progressive convergence of cost function among the iterations when we use BGD-based models. In summary, Table 2 outlines the optimal (minimum) signaling overhead ratio required for the all proposed system configurations, the optimal model (holding the smallest total cost function), the related combination of optimal hyperparameters, and the corresponding train/test error values. When the signal is affected with much noise, it is harder to keep the same range of error when compared with high a $P_{u}$ regime. In fact, MF models keep the same (minimum) signaling overhead (0.1) regardless of the transmitted power regime, being able to accurately predict with just 10% of sounded beams. Thus, the proposed MF/NMF methods are able to reduce the pilot signaling overhead by 90% compared with Exhaustive BA with negligible training and test errors.

## 5. Multi-Layer Perceptron

### 5.1. MLP Problem Formulation

**Figure 6:**
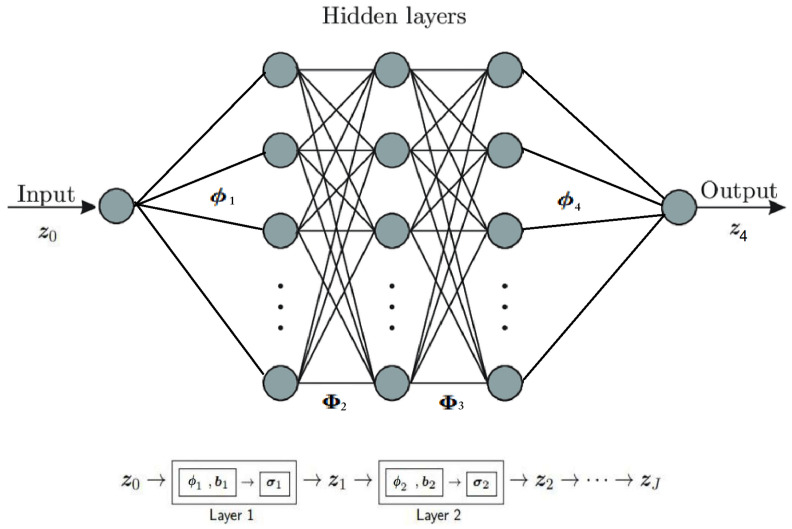
Multi-Layer Perceptron architecture (toy example with J=4).

We consider a feed-forward MLP, with *J* layers, modeled as a composition of *J* non-linear functions/layers. Let $z_{0}\in R$ be the MLP input, and $z_{J}\in R$ be the MLP output; see Figure 6. We denote with $\left\{z_{2},\ldots ,z_{J-1}\right\}$ all the hidden layers. We assume for simplicity that the width of all the layers is the same, denoted as *D*, i.e., $\left\{z_{2}\in R^{D},\ldots ,z_{J-1}\in R^{D}\right\}$; see Figure 6. The equation describing layer 1 is given by $z_{1}=\sigma_{1}\left(\phi_{1}z_{0}\right)=\sigma_{1}\left(\phi_{1}1\right)$, where $z_{1}\in R^{D}$ is the output of layer 1, $\phi_{1}\in R^{D}$ is the resulting weight vector, and $\sigma_{1}\left(\right):R⟶R^{D}$ is the non-linear activation function for layer 1. We use one hot encoding for the MLP input $z_{0}\in R$, i.e., $z_{0}=1$ for all training samples, ∀(u,i)∈K. We express the output of the hidden layers, ${\left\{z_{j}\in R^{D}\right\}}_{j=2}^{J-1}$, as $z_{j}=\sigma_{j}\left(\Phi_{j}z_{j-1}\right)\;,\;\forall \;j\in \left\{2,\ldots ,J-1\right\}$, where $z_{j-1}\in R^{D}$ is the input of the layer *j* and $z_{j}\in R^{D}$ is its output ,∀j∈{2,…,J−1}; $\Phi_{j}\in R^{D\times D}$ is the weight matrix for the layer *j*,∀j∈{2,…,J−1}; and $\sigma_{j-1}\left(\right):R^{D}⟶R^{D}$ is the element-by-element non-linear activation function for the layer *j*, ∀j∈{2,…,J−1}. Finally, the relation for the last layer j=J is expressed as $z_{J}=\sigma_{J}\left(\phi_{J}z_{J-1}\right)$, where $z_{J}\in R$ is the output for layer *J*, $\phi_{J}\in R^{1\times D}$ is its weight vector, and $\sigma_{J}\left(\right):R^{D}⟶R$ is the non-linear activation function for the layer *J*. We express the output of the MLP $z_{J}\in R$ (as a function of all layers) as

(16) $$\begin{array}{c} z_{J}:=\sigma_{J}\left(\phi_{J}\ldots \sigma_{2}\left(\Phi_{2}\left(\sigma_{1}\left(\phi_{1}\right)\right)\right)\right) \end{array}$$

The output of MLP is made to fit/approximate all the RSE values at all training samples; $z_{J}:=RSE_{u,i}$, ∀(u,i)∈K. We define the MSE loss $l_{u,i}$ for the sample (u,i) in the training set K as the distance between the MLP output $z_{J}$ and the known RSE label for the beam pair (u,i), ${\mathrm{RSE}}_{u,i}$, i.e,

$$\begin{array}{c} l_{u,i}:={\left(z_{J}-RSE_{u,i}\right)}^{2}={\left(\;\underset{MLP\;output}{\underset{︸}{\sigma_{J}\left(\phi_{J}\ldots \sigma_{2}\left(\Phi_{2}\left(\sigma_{1}\left(\phi_{1}\right)\right)\right)\right)}}-\underset{RSE\;value}{\underset{︸}{RSE_{u,i}}}\right)}^{2}\;,\;\forall \left(u,i\right)\;\in K \end{array}$$

Then, the empirical risk is defined as the average of the individual loss $l_{u,i}$ across the training set K, $\left(1/|K|\right)\sum_{(u,i)\in K}l_{u,i}$. The empirical risk minimization for the MLP is given in (P3).

$$\begin{array}{c} \left(P3\right):=\{\left({\phi_{1}}^{*},{\Phi_{2}}^{*},\ldots ,{\phi_{J}}^{*}\right)\left\{\begin{array}{c} \underset{\phi_{1},\Phi_{2},\ldots ,\Phi_{J-1},\phi_{J}}{argmin}\;\frac{1}{\left|K\right|}\sum_{(u,i)\in K}\;l_{u,i}\left(\phi_{1},\Phi_{2},\ldots ,\Phi_{J-1},\phi_{J}\right)\\ s.t.\;\phi_{1}\;\in \;R^{D},\Phi_{2}\;\in \;R^{D\times D},\ldots ,\;\Phi_{J-1}\;\in \;R^{D\times D},\;\phi_{J}\;\in \;R^{1\times D} \end{array}\right. \end{array}$$

### 5.2. MLP Learning

We propose to learn the optimal MLP weights via back-propagation (BP). We choose an arbitrary mini-batch of samples of size B⊆K and define the mini-batch loss as

(17) $$\begin{array}{c} l_{B}:=\frac{1}{\left|B\right|}\sum_{u,i\in B}{\left(\sigma_{J}\left(\phi_{J}\ldots \sigma_{2}\left(\Phi_{2}\left(\sigma_{1}\left(\phi_{1}\right)\right)\right)\right)-RSE_{u,i}\right)}^{2}\;,\;\forall \;\left(u,i\right)\in B \end{array}$$

We express the partial derivative of the loss corresponding to the mini-batch $l_{B}$ with respect to each layer $\Phi_{j},j\left\{1,\ldots ,J\right\}$ as

(18) $$\begin{array}{c} \frac{\partial l_{B}}{\partial \Phi_{j}}=\frac{1}{\left|B\right|}\sum_{(u,i)\in B}\left(\delta_{j}{z_{j-1}}^{T}\;\right),\;\forall j\in \left\{1,..J\right\}\;, \end{array}$$

where

$$\begin{array}{c} \delta_{j}\overset{\Delta }{=}\left\{\begin{array}{c} \left({\Phi_{j+1}}^{T}\delta_{j+1}\right)\circ \sigma_{j}^{\prime },j<J\\ 2\left(z_{J}-RSE_{u,i}\right)\circ \sigma_{j}^{\prime },j=J\;,\;\left(u,i\right)\in B \end{array}\right.,\sigma_{j}^{\prime }\overset{\Delta }{=}\frac{\partial \sigma (u)}{\partial u}={\left[\frac{\partial \sigma (u_{1})}{\partial u_{1}},\ldots ,\frac{\partial \sigma (u_{d_{j}})}{\partial u_{d_{j}}}\right]}^{T}, \end{array}$$

j=1,…,J and ∘ denotes the Hadamard product. We express the BP weight update of the mini-batch loss $l_{B}$, for all layers ∀j∈{1,…,J}, as

(19) $$\begin{array}{c} \Phi_{j}^{\left(k+1\right)}=\Phi_{j}^{\left(k\right)}-{\beta_{j}}^{\left(k\right)}\frac{\partial l_{B}}{\partial \Phi_{j}}{|}_{\Phi_{j}^{\left(k\right)}},\forall j\in \left\{1,\ldots ,J\right\},\;k=\left\{1,\ldots ,T\right\} \end{array}$$

where ^(*k*)^ is the BP iteration index, $\Phi_{j}^{\left(k\right)}$ is the value of $\Phi_{j}$ at iteration *k*, ${\beta_{j}}^{\left(k\right)}$ is the BP step size (learning rate) for the layer *j* at iteration *k*, and $\frac{\partial l_{B}}{\partial \Phi_{j}}{|}_{\Phi_{j}^{\left(k\right)}}$ is the partial derivative given in (18) evaluated at $\Phi_{j}^{\left(k\right)}$.

**Back-propagation algorithm with mini-batch**

Choose the mini-batch B as a random subset of the training set K.

1. Compute the loss function $l_{B}$ for all samples in the mini-batch (u,i)∈B in (17).
2. Compute the partial derivative $\frac{\partial l_{B}}{\partial \Phi_{j}}$ of the mini-batch loss $l_{B}$ with respect to $\Phi_{j}$ in (18).
3. Update the weights of each layer as in (19).

We assume that the BP learning rate is the same for all layers, $\beta_{j}^{\left(k\right)}=\beta^{k},\forall j\in \left\{1,\ldots ,J\right\}$.

### 5.3. Prediction Using MLP

The MLP prediction for the sample (u,i) in the test set L, using optimal weights ${\phi_{1}}^{*}$, ${\Phi_{2}}^{*},\ldots ,{\phi_{J}}^{*}$ is as follows:

(20) $$\begin{array}{c} \hat{z_{J}}=\sigma_{J}\left({\phi_{J}}^{*}\ldots \sigma_{2}\left({\Phi_{2}}^{*}\left(\sigma_{1}\left({\phi_{1}}^{*}\right)\right)\right)\right),\forall \left(u,i\right)\in L \end{array}$$

Therefore, the test MSE is defined as

(21) $$\begin{array}{c} \frac{1}{\left|L\right|}\sum_{(u,i)\in L}\;{\left(\;\hat{RSE_{u,i}}-\sigma_{J}\left({\phi_{J}}^{*}\ldots \sigma_{2}\left({\Phi_{2}}^{*}\left(\sigma_{1}\left({\phi_{1}}^{*}\right)\right)\right)\right)\;\right)}^{2} \end{array}$$

We then select the optimal indexes $u^{★}$ and $i^{★}$ related to the highest $RSE_{u,i}$ value, as follows:

(22) $$\begin{array}{c} \left(u^{★},i^{★}\right)=argmax_{(u,i)\in L\cup K}\;\;\left\{RSE_{u,i}|\forall \left(u,i\right)\in K\right\}\cup \left\{\hat{RSE_{u,i}}|\forall \left(u,i\right)\in L\right\} \end{array}$$

### 5.4. Proposed BA Algorithm Using MLP

The Multi-Layer Perceptron-based Beam Alignment is specified in Algorithm 2.
**Algorithm 2** Proposed MLP-Based BA Method.Input: ${\left\{f_{u}\right\}}_{\forall u\in T}$, ${\left\{W_{i}\right\}}_{\forall i\in R}$, η, Pu-Generate randomly sub-sampled codebooks, $T_{S},R_{S}$, satisfying $\left(\right|T_{S}\left|.\right|R_{S}\left|\right)/\left(|T\right|\times \left|R|\right)=\eta$-Sound beam pairs from training set, $K:=T_{S}\times R_{S}$.-Record corresponding RSE and generate RSE mat. S, in (5)-Train MLP weights (using back-propagation algorithm)
return optimal weights, $\left\{{\phi_{1}}^{*},{\Phi_{2}}^{*},\ldots ,{\phi_{J}}^{*}\right\}$
-Use optimal parameters $\left\{{\phi_{1}}^{*},{\Phi_{2}}^{*},\ldots ,{\phi_{J}}^{*}\right\}$, to predict unknown RSE of test set, L, in (21)-Search training and test sets, for optimal beam pair $\left(u^{★},i^{★}\right)$, holding the largest RSE, (22)Output: $f_{u^{★}}$, $W_{i^{★}}$

We assume that the number of neurons per layer *D*, the number of layers *J*, the mini-batch size B=|B|, and the BP learning rate $\beta^{\left(k\right)}$ are hyperparameters. They are tuned using a grid search cross-validation.

### 5.5. Numerical Simulations

We define the training and test cost functions as follows:

(23) $$\begin{array}{c} Train\;NMSE=\frac{1}{\left|K\right|}\left(\sum_{(u,i)\in K}{\left(\frac{RSE_{u,i}-\sigma_{J}\left(\phi_{J}\ldots \sigma_{2}\left(\Phi_{2}\left(\sigma_{1}\left(\phi_{1}\right)\right)\right)\right)}{RSE_{u,i}}\right)}^{2}\right) \end{array}$$

(24) $$\begin{array}{c} Test\;NMSE=\frac{1}{\left|L\right|}\left(\sum_{(u,i)\in L}{\left(\frac{\hat{RSE_{u,i}}-\sigma_{J}\left({\phi_{J}}^{*}\ldots \sigma_{2}\left({\Phi_{2}}^{*}\left(\sigma_{1}\left({\phi_{1}}^{*}\right)\right)\right)\right)}{\hat{RSE_{u,i}}}\right)}^{2}\right) \end{array}$$

Therefore, we used the same system configurations as for MF/NMF, resumed in Table 1. Moreover, we choose the learning rate $\beta_{k}\in \{0.1$, 0.01, 0.001, 0.0001}, while the batch size B∈{2, 4, 8, 16, 32, 64, 128}, the number of hidden layers J∈{1,2,3}. For each layer, the number of neurons D∈{8, 16, 32, 64, 128}. We use the Rectified Linear Units as our activation function for all layers.

**Figure 7:**
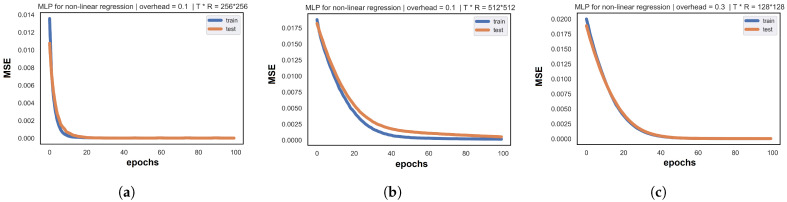
MLP Learning curves: (**a**) learning curve: 256 × 256 with overhead 0.1 MLP; (**b**) learning curve: 512 × 512 with overhead 0.1 MLP; and (**c**) learning curve: 128 × 128 with overhead 0.3 MLP.

Similar to MF/NMF, train performance is observed when we track the evolution of the cost function NMSE, applied to the training samples of the set K, in a function of iterations. The range of considerably low-error values and the overall learning behavior of the MLP architecture illustrates that our shallow neural network successfully resolves the non-linear regression problems related to our BA process. For massive setups, MLP reaches around $10^{-6}$ error in a high $P_{u}$ regime. However, this cost value increases as long as the amount of noise and interference augments. Note that the train NMSE also decreases when we increase the size of the dataset matrix S, which provides more samples for MLP to improve the feature extraction and the prediction quality. Regarding the unknown beams, test error values in the numerical result tables are close to the train cost (with no overfitting or underfitting in the corresponding learning curves). Moreover, the test loss is impacted by the transmitted power regime the same way as the training process. Identical to GD-based MF/NMF, the MLP learning curves in Figure 7 plot the same shape of curve with a continuous monotonic decrease in the train and test cost among the iterations: the convergence is progressive among the iterations, and at the last epoch, training and test NMSE values land at considerably low error values and prove that MLP accurately fits to our problem and provides a concrete solution for ML-based BA. From a QoS perspective, Table 3 resumes the smallest (optimal) signaling overhead required for a successful beam sounding based on reliable prediction quality. Similar to MF/NMF, for all the proposed transmitted power, MLP requires 10% of the total beam pairs to fulfill the RSE matrix.

## 6. Results and Discussion

### 6.1. Train/Test Prediction Performance Comparison

For the six MF-based models, we select the best one (minimum test error) to represent the MF family of methods in this section and compare it with MLP. When we analyze QoS (Table 1 and Table 2), we notice that the transmitted power regime impacts the quality of prediction by reducing the overall loss. For MF/NMF, we observe that the loss damage is large. We jump from around $10^{-8}$ for massive configurations (256, 512, and 1024) to $10^{-4}$ for smaller setups. For MLP, we spot the increase in the overall loss when we decrease $P_{u}$. Thus, MLP seems to be the most robust architecture with respect to changing the transmitted power. Additionally, we empirically notice that the change in the $P_{u}$ values does not impact the optimal hyperparameters selected from cross-validation. Furthermore, when we track the evolution of the training/test cost in the function of iterations, we observe balanced models with no signs of overfitting or underfitting. On the other hand, when the transmitted power decreases, MF/NMF tend to be the most impacted models in terms of train/test error, while the MLP error is robust.

On the other hand, from a QoS perspective, concerning the evolution of the optimal (minimum) required signaling overhead and what impact can the $P_{u}$ regime have on the optimal required values, in reference to Table 1 and Table 2, all the proposed models required just 10% of the total number of beam pairs at UE and BS for all antenna configurations from 128×128 to 1024×1024 for all the proposed Pu values. This proves that the transmitted power impacts the quality of prediction but not the number of beam pairs required for training. In fact, low $P_{u}$ leads to damaging the signal quality and subsequently damages the quantity of useful information to be extracted from the datasets. Finally, the only cases where the $P_{u}$ regime impacts the optimal overhead ratio is among the smallest configurations, for instance, the 16×16 setup where it seems normal for all learning models to demand more data to learn from (more hidden interactions between UE and BS as features to extract). These are the experimental situations where Exhaustive BA is technically feasible.

### 6.2. Similarities and Differences between Models

**Figure 8:**
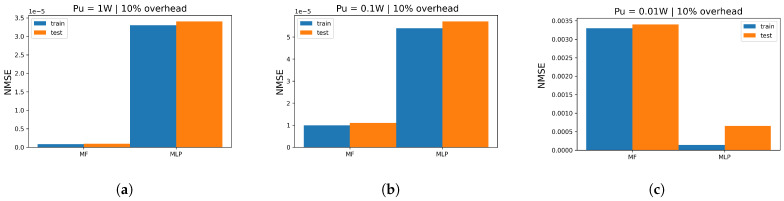
Train/test NMSE in function of $P_{u}$ for all proposed models for 512×512 using optimal overhead ratio; (**a**) 512 × 512 train/test NMSE for $P_{u}=1$ W; (**b**) 512 × 512 train/test NMSE for $P_{u}=10^{-1}$ W; (**c**) 512 × 512 train/test NMSE for $P_{u}=10^{-2}$ W.

**Figure 9:**
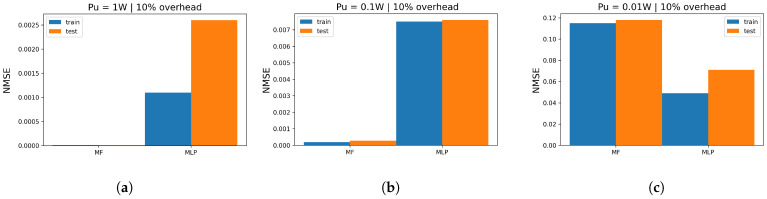
Train/test NMSE in function of $P_{u}$ for all proposed models for 128×128 using optimal overhead ratio: (**a**) 128 × 128 train/test NMSE for $P_{u}=1$ W; (**b**) 128 × 128 train/test NMSE for $P_{u}=10^{-1}W$; (**c**) 128 × 128 train/test NMSE for $P_{u}=10^{-2}$ W.

**Figure 10:**
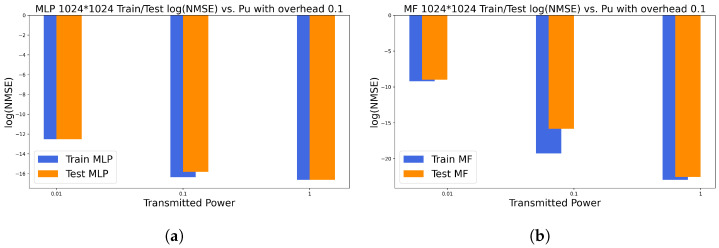
Log(NMSE) in function of $P_{u}$ for 1024×1024 using optimal overhead ratio: (**a**) MLP train/test log(NMSE) in function of Pu using optimal overhead ratio; (**b**) MF train/test log(NMSE) in function of $P_{u}$ using optimal overhead ratio.

All models required just 10% of the beams for training for all the proposed massive setups. Moreover, all the proposed models are shallow neural architectures with few hidden layers for low-complexity constraints. Even among the largest configurations, the optimal dimensions of models picked from the cross-validation illustrate small networks with no need to require dense architectures. Furthermore, all models succeeded with the matrix completion task, and they all illustrate a monotonic decrease in loss values as long as we increase the MIMO setup. Additionally, MF-based models are the most accurate reaching loss values in the range $10^{-8}$ for massive setups in a high $P_{u}$ regime, and their cross-validation illustrates smaller grid search where there are fewer hyperparameters to tune. However, they are the slowest models when applied to high-dimensional MIMO setups. On the other hand, MLP illustrates a good balance between run time (complexity) and loss values (prediction quality). It reaches around $10^{-4}$ and $10^{-5}$ loss for massive configurations. In addition, the MLP is the most robust model facing the changes in the $P_{u}$ values. In Figure 8, for 512×512, the figure illustrates the train/test NMSE in the function of each model and the corresponding transmitted power: in Figure 8a, for $P_{u}=1W$, MF achieves its best performance, slightly better than MLP with the difference between achieved cost values at around $10^{-1}$. In Figure 8b, when $P_{u}=10^{-1}W$, MF still gets the best performance, marginally better than MLP with an NMSE value difference of around $10^{-1}$. In Figure 8c, when $P_{u}=10^{-2}$, MF noticeably gets impacted (overall loss around $10^{-3}$) while MLP provides the best prediction performance: this suggests that when $P_{u}$ is small, MLP is more robust than MF/NMF, which performs best in high $P_{u}$ regime. Similarly, almost same remarks hold for Figure 9 when we simulate the 128×128 configuration: in Figure 9a, MF reaches considerably better performance compared with MLP with $10^{-4}$. In Figure 9b, MLP kept the same range of error, which states again the robustness of the model while MF got severely impacted ($10^{-3}$) but sill holds the best performance. In Figure 9c, when $P_{u}$ is weak, MF illustrates the worst performance in all simulations. On the other hand, MLP got slightly impacted with an overall loss of $10^{-1}$ and reaches the best quality of prediction. In Figure 10, we investigate the highest configuration 1024×1024. Similar conclusions for Figure 8 and Figure 9 hold for this figure in terms of best model (MF for $P_{u}=1W$, $P_{u}=10^{-1}$ and MLP for $P_{u}=10^{-2}$). In addition, we aim to investigate the overall impact of varying the transmitted power. Thus, we track the log(NMSE) values while switching from one $P_{u}$ regime to another: In Figure 10, in Figure 10a, for MLP, the curve gap from low/medium is $log{\left(NMSE\right)}_{medium}-log{\left(NMSE\right)}_{low}\approx -16-\left(-12\right)\approx -4$. The gap in the medium/high regimes is almost negligible ( $log{\left(NMSE\right)}_{high}-log{\left(NMSE\right)}_{medium}\approx -16-\left(-16\right)\approx 0.5$). Finally, in Figure 10b, the MF gap is around $log{\left(NMSE\right)}_{medium}-log{\left(NMSE\right)}_{low}\approx -17-\left(-9\right)\approx -8$ and $log{\left(NMSE\right)}_{high}-log{\left(NMSE\right)}_{medium}\approx -22-\left(-17\right)\approx -5$: at each change of $P_{u}$, MF is considerably impacted. To sum up, the choice of the optimal model strongly depends on the available complexity and the given transmitted power $P_{u}$. In fact, MF, whether through BCD or BGD optimization, is the best model when the transmitted power is high ($P_{u}=1W$). In this case, BCDMF converges faster but has higher complexity than BGD. However, SGD for MF/NMF are the slowest models to converge but show negligible complexity. On the other hand, if we aim to prioritize run time, MLP exhibits the fastest predictions with good prediction error. Finally, it is wise to opt for MLP if the system is to operate under various transmitted power regimes where MLP offers good prediction quality for every $P_{u}$ value and the available complexity is medium.

## 7. Conclusions

In this paper, we proposed a blind Machine Learning-based Beam Alignment using Matrix Factorization, non-negative Matrix Factorization, and Multi-Layer Perceptron. We assumed an Uplink massive mmWave MIMO system using single RF-chains at UE and multiple RF-chains at BS though a fully analog architecture. The proposed approach consists in sounding the RSE of sub-sampled codebooks at UE and BS. The RSE of the non-sounded beams is predicted using MF, NMF, and MLP models. Our results show that, by sounding just 10% of the total beam pair samples, we may predict with high accuracy the unknown RSE values, which massively reduce the large signaling overhead of Exhaustive BA. Our future work investigates the scalability of our approach to a multi-user scenario. Robustness and ML-interpretability are other research directions for modeling industrial deployment.

## Figures and Tables

**Table 1:** System parameters and hyperparameters.

System Configuration for All Proposed Models	
System parameter	Numerical value
number of antennas $N_{T}$ at UE	128,256,512,1024
number of antennas $N_{R}$ at BS	128,256,512,1024
codebook cardinality |T| at UE	128,256,512,1024
codebook cardinality |R| at BS	128,256,512,1024
overhead ratio η regime	0.7,0.5,0.3,0.1
number of OFMD sub-carriers $N_{c}$	64
number of channel paths *L*	2 (NLoS)
transmitted power $P_{u}$ (W)	$1,10^{-1},10^{-2}$
MF/NMF dimension $D_{MF}$	2,3,4,5,6
MF/NMF learning rate $\alpha_{k}$	$10^{-1},10^{-2},10^{-3},10^{-4},10^{-5},10^{-6}$
MF/NMF regularization factors λ,μ	$10^{-2},10^{-3},10^{-4},10^{-5},10^{-6},10^{-7}$
MLP number of layers *J*	1,2,3
MLP number of neurons per layer $D_{MLP}$	8,16,32,64,128
MLP batch size *B*	2,4,8,16,32,64,128
MLP learning rate $\beta_{k}$	$10^{-1},10^{-2},10^{-3},10^{-4}$

**Table 2:** QoS minimum overhead required for MF/NMF for all proposed Pu regimes.

a | MF/NMF | QoS Minimum Overhead Required for $P_{u}=1$ W				
MIMO setup	Optimal hyperparameters	Min Overhead	Train NMSE	Test NMSE
128 by 128	BGD NMF{D = 2, (λ, μ) = (0.0001, 0.0001), $\alpha_{k}$ = 0.001}	0.1	8.407746 $\times \;10^{-6}$	9.147875 $\times \;10^{-6}$
256 by 256	BGD MF{D = 3, (λ, μ) = (0.0001, 0.0001), $\alpha_{k}$ = 0.001}	0.1	4.102708 $\times \;10^{-6}$	7.344720 $\times \;10^{-6}$
512 by 512	BGD MF{D = 4, (λ, μ) = (0.0001, 0.0001), $\alpha_{k}$ = 0.001}	0.1	8.374633 $\times \;10^{-7}$	9.417057 $\times \;10^{-7}$
1024 by 1024	SGD NMF{D = 4, (λ, μ) = (0.0001, 0.0001), $\alpha_{k}$ = 0.01}	0.1	1.219227 $\times \;10^{-7}$	1.616363 $\times \;10^{-7}$
**b | MF/NMF | QoS Minimum Overhead Required for $P_{u}=10^{-1}$ W**				
MIMO setup	Optimal hyperparameters	Min Overhead	Train NMSE	Test NMSE
128 by 128	SGD NMF {D = 2, (λ, μ) = (0.0001, 0.0001), $\alpha_{k}$ = 0.001}	0.1	0.000191	0.000276
256 by 256	SGD NMF {D = 3, (λ, μ) = (0.0001, 0.0001), $\alpha_{k}$ = 0.001}	0.1	4.648861 $\times \;10^{-5}$	5.775554 $\times \;10^{-5}$
512 by 512	BGD NMF{D = 4, (λ, μ) = (0.0001, 0.0001), $\alpha_{k}$ = 0.001}	0.1	1.052556 $\times \;10^{-5}$	1.170430 $\times \;10^{-5}$
1024 by 1024	BGD NMF {D = 4, (λ, μ) = (0.0001, 0.0001), $\alpha_{k}$ = 0.001}	0.1	1.600790 $\times \;10^{-6}$	1.695907 $\times \;10^{-6}$
**c | MF/NMF | QoS Minimum Overhead Required for $P_{u}=10^{-2}$ W**				
MIMO setup	Optimal hyperparameters	Min overhead	Train NMSE	Test NMSE
128 by 128	SGD MF {D = 2, (λ, μ) = (0.0001, 0.0001), $\alpha_{k}$ = 1 $\times \;10^{-6}$}	0.1	0.115517	0.118776
256 by 256	BGD MF {D = 3, (λ, μ) = (0.0001, 0.0001), $\alpha_{k}$ = 0.0001}	0.1	0.016475	0.016679
512 by 512	SGD NMF{D = 4, (λ, μ) = (0.0001, 0.0001), $\alpha_{k}$ = 1 $\times \;10^{-6}$}	0.1	0.003371	0.003449
1024 by 1024	BGD MF {D = 4, (λ, μ) = (0.0001, 0.0001), $\alpha_{k}$ = 1 $\times \;10^{-5}$}	0.1	0.001681	0.001948

**Table 3:** QoS minimum overhead required for MLP for all the proposed $P_{u}$ regimes.

a | MLP | QoS Minimum Overhead Required for $P_{u}=1$ W				
MIMO setup	Optimal hyperparameters	Min overhead	Train NMSE	Test NMSE
128 by 128	{(J = 3, D = 8), B = 4, $\beta_{k}$ = 0.0001}	0.1	0.001144	0.002639
256 by 256	{(J = 3, D = 16), B = 16, $\beta_{k}$ = 0.001}	0.1	3.941522 $\times \;10^{-5}$	3.948157 $\times \;10^{-6}$
512 by 512	{(J = 3, D = 64), B = 32, $\beta_{k}$ = 0.0001}	0.1	3.305507 $\times \;10^{-5}$	3.335168 $\times \;10^{-5}$
1024 by 1024	{(J = 3, D = 64), B = 64, $\beta_{k}$ = 0.0001}	0.1	9.810028 $\times \;10^{-6}$	9.857067 $\times \;10^{-6}$
**b | MLP | QoS Minimum Overhead Required for $P_{u}=10^{-1}$ W**				
MIMO setup	Optimal hyperparameters	Min overhead	Train NMSE	Test NMSE
128 by 128	{(J = 3, D = 8), B = 4, $\beta_{k}$ = 0.0001}	0.1	0.007569	0.007662
256 by 256	{(J = 3, D = 16), B = 16, $\beta_{k}$ = 0.001}	0.1	0.000139	0.000288
512 by 512	{(J = 3, D = 64), B = 32, $\beta_{k}$ = 0.0001}	0.1	5.419598 $\times \;10^{-5}$	5.756302 $\times \;10^{-5}$
1024 by 1024	{(J = 3, D = 64), B = 64, $\beta_{k}$ = 0.0001}	0.1	1.184073 $\times \;10^{-5}$	1.72301 $\times \;10^{-5}$
**c | MLP | QoS Minimum Overhead Required for $P_{u}=10^{-2}$ W**				
MIMO setup	Optimal hyperparameters	Min overhead	Train NMSE	Test NMSE
128 by 128	{(J = 3, D = 8), B = 4, $\beta_{k}$ = 0.0001}	0.1	0.049559	0.071185
256 by 256	{(J = 3, D = 16), B = 16, $\beta_{k}$ = 0.001}	0.1	0.017011	0.017634
512 by 512	{(J = 3, D = 64), B = 32, $\beta_{k}$ = 0.0001}	0.1	0.000141	0.000666
1024 by 1024	{(J = 3, D = 64), B = 64, $\beta_{k}$ = 0.0001}	0.1	1.700140 $\times \;10^{-4}$	1.702889 $\times \;10^{-4}$

## Data Availability

Datasets are available from the authors upon reasonable request.

## Abbreviations

ALS	Alternating Least Squares
AoD	Angle of Departure
AoA	Angle of Arrival
AWGN	Additive White Gaussian Noise
BA	Beam Alignment
BS	Base Station
BCE	Binary Cross Entropy
BCD	Block Coordinate Descent
BGD	Block Gradient Descent
BSGD	Block Stochastic Gradient Descent
CSI	Channel State Information
DFT	Discrete Fourier Transform
GD	Gradient Descent
LoS	Line of Sight
MF	Matrix Factorization
MIMO	Multiple Input Multiple Output
ML	Machine Learning
MLP	Multi-Layer Perceptron
MSE	Mean Squared Error
NMF	Non-Negative Matrix Factorization
NLoS	Non Line of Sight
NMSE	Normalized Mean Squared Error
OFDM	Orthogonal Frequency Division Multiplexing
QoS	Quality of Service
ReLu	Rectified Linear Unit
RSE	Received Signal Energies
SNR	Signal-to-Noise Ratio
UE	User Equipment

## Appendix A. Proof: BCD Convergence

We will show that the two (below) necessary conditions for convergence of BCD are satisfied:

(i) The loss function is strongly convex, per block; i.e., we need to show that sub-problem S1 and S2 have a unique solution.
(ii) The constraints of the MF prob $\theta_{u}\in R^{d}\;,\;\psi_{i}\in R^{d}\;$, are separable and individually convex.

Recall that sub-problem S1 is written as

$$\begin{array}{cc} \left(S1\right):{\theta_{u}}^{\left(k+1\right)} & =argmin_{\theta_{u}\in R^{d}}\;\;\left[-2{\theta_{u}}^{T}{r_{u}}^{\left(k\right)}+{\theta_{u}}^{T}\left({Q_{u}}^{\left(k\right)}+\mu_{u}I_{D}\right)\theta_{u}\right]\;=\;f_{1}\left(u\right),\;\;\forall u, \end{array}$$

Next, we will prove that the equivalent form in (S1), is a strongly convex function; i.e., it shows that $f_{1}\left(\theta_{u}\right)$ is strongly in $\theta_{u}$. To that end, we derive the corresponding Hessian:

$$\begin{array}{cc} \nabla^{2}f_{1}\left(\theta_{u}\right) & :=2(\;Q_{u}^{\left(k\right)}+\mu_{u}I_{D}\;),\;\;\forall u, \end{array}$$

For this Hessian expression, $Q_{u}^{\left(k\right)}⪰0$ is a Positive Semi Definite (PSD) matrix (by def), $\mu_{u}I≻0$ is a Positive Definite (PD) matrix, and $(\;Q_{u}^{\left(k\right)}+\mu_{u}I_{D}\;)≻0$ is a PD matrix. Thus, the Hessian is a PD matrix $\nabla^{2}f_{1}\left(\theta_{u}\right)≻0$, and $f_{1}\left(\theta_{u}\right)$ is strongly in $\theta_{u}$, and the solution to the sub-problem (S1) is unique. Recall that the sub-problem (S2) is expressed as

$$\begin{array}{c} \left(S2\right):{\psi_{i}}^{\left(k+1\right)}=argmin_{\psi_{i}\in R^{d}}\;\left[-2t_{i}^{{\left(k+1\right)}^{T}}\psi_{i}+{\psi_{i}}^{T}\left(\;P_{u}^{\left(k+1\right)}+\lambda_{i}I\;\right)\psi_{i}\right]=f_{2}\left(\psi_{i}\right)\;,\;\forall i\;, \end{array}$$

Next, we will prove that the equivalent form is a strongly convex function; i.e., it shows that $f_{2}\left(\psi_{i}\right)$ is strongly in $\psi_{i}$. To that end, we derive the corresponding Hessian:

$$\begin{array}{c} \nabla^{2}f_{2}\left(\psi_{i}\right):=2\left(\;P_{i}^{\left(k+1\right)}+{\lambda_{i}}^{\left(i\right)}I_{D}\;\right),\;\;\forall i, \end{array}$$

For this Hessian expression, $P_{i}^{\left(k+1\right)}⪰0$ is a PSD matrix (by def), $\lambda_{i}^{\left(i\right)}I≻0$ is a PD matrix, and $(\;P_{i}^{\left(k+1\right)}+\lambda_{i}^{\left(i\right)}I_{D}\;)≻0$ is a PD matrix. Thus, the Hessian is a PD matrix $\nabla^{2}f_{2}\left(\psi_{i}\right)≻0$, and $f_{2}\left(\psi_{i}\right)$ is strongly convex in $\psi_{i}$. Thus, the solution to the sub-problem (S2) is unique.
